# Isolation and Identification of Yeasts in Marcha, a Rice Wine Starter Culture From Nepal

**DOI:** 10.1155/2024/4188578

**Published:** 2024-09-16

**Authors:** Jayram Karmacharya, Prasansah Shrestha, Tika Bahadur Karki, Om Prakash Pant

**Affiliations:** ^1^ Department of Microbiology National College (NIST) Tribhuvan University, P.O. Box 8659, Naya Bazar, Kathmandu, Nepal; ^2^ Department of Food Technology Central Campus of Technology Tribhuvan University, Dharan, Nepal

**Keywords:** ethanol production attributes, Marcha, stress exclusion tests, yeasts

## Abstract

Nepal harbors a rich diversity of cultures and traditions, including the unique practice of creating an indigenous grain called Marcha by various ethnic groups such as Newar, Tamang, Sherpa, Rai, Limbu, Gurung, Magar, and Tharu people. In the eastern region of Nepal, Marcha producers utilize over 42 different plants, including *Vernonia cinerea*, *Clematis grewiae*, *Polygala arillata*, *Buddleja asiatica*, *Inula* sp., *Scoparia*, and more, which shows regional diversity. The primary objective of the study was to explore the diversity of yeast present in Marcha samples. The studied Marcha samples were collected from 10 different geographic regions of Nepal, which included altogether 27 samples. The isolates were grouped into Groups A, B, and C based on morphological and physiological characteristics. Notably, Group B yeast displayed high amylase production, an enzyme responsible for starch breakdown, and exhibited the ability to produce ethanol. To further investigate the potential of these isolates, stress exclusion tests were conducted, with 30 isolates (70%) showing positive responses. The yeast isolates demonstrated resilience to high glucose concentrations of up to 36% (*w*/*v*) at a pH above 3 and a temperature of 37°C, which is the ideal growth condition. The study observed a direct correlation between the yeast isolates' ethanol production capabilities and their tolerance to different ethanol concentrations. Considering that all tested Marcha samples contained yeast capable of starch degradation and ethanol production, it was expected that these yeast isolates would actively participate in the fermentation of starch-based alcohol.

## 1. Introduction

In oriental countries, people use microbial inoculum made from starchy cereals, either in the form of dry powder or hard balls, to initiate food and alcohol fermentation. These starters are known by different names across the region, such as Murcha or Marcha in the Himalayan regions of Nepal, India, and Bhutan; ragi in Indonesia; loog-pang in Thailand; bubod in the Philippines; Chinese yeast in Taiwan [1]; and nuruk in Korea [2]. Marcha, specifically, is a mixed dough inoculate employed as a starter culture to produce various traditional alcoholic beverages like jaanr and raksi in Nepal, India, and Bhutan [3]. It is a solid ball-like structure, dry, and round-to-flattened in shape. It comes in creamy white to dusty white colors and can vary in size, ranging from 1.9 to 11.8 cm in diameter and weighing between 2.3 and 21.2 g. The dialects used by many ethnic groups in the area vary, with the Limboo calling it khesung, the Tamang bharama, the Rai bopkha or khabed, the Bhutia and Tibetans phab, and the Lepcha buth/thanbum. It has been a long-standing custom to produce alcohol products through spontaneous fermentation, which is triggered by local yeasts of various genera and species [4–7]. Several factors influence the number of species and their presence during fermentation [8, 9].

Marcha contains various microorganisms, including filamentous molds like *Mucor circinelloides* and *Rhizopus chinensis*, yeasts such as *Saccharomycopsis fibuligera* and *Pichia anomala*, and bacteria such as *Pediococcus pentosaceus* [3]. Although alcohol-producing bacteria have not yet been found, the presence of saccharifying yeast species from the genus *Saccharomyces* suggests their involvement in amylolytic fermentation [3, 10].

To produce starter strains that may be able to generate the customary flavor and aroma as well as to ensure the conservation of gene pools of technological significance, it is essential to maintain biological patrimony. Therefore, exploring the diversity of native fermentative strains helps us understand and select strains with specific characteristics. Among the diverse group of microorganisms, yeasts were chosen due to their coexisting role in Marcha for the formation of amylase and alcohol [11]. On the other hand, the demand for new yeast strains has grown in various industries, such as ethanol production for energy, solvents, cleansing agents, and preservatives, and has increased, leading to the need for higher production. With a global shift towards ethanol production through fermentation instead of chemical synthesis from petrochemical substrates, it becomes crucial to identify well-suited microbial strains, appropriate fermentation substrates, and suitable process technology to maximize ethanol yield. According to Stewart et al. [12], an ideal microbe for ethanol production should possess rapid fermentation potential, good flocculating ability, notable osmotolerance, enhanced ethanol tolerance, and strong thermotolerance.

However, most industrial microorganisms are patented and may not be usable outside of their country of origin due to the proprietary nature of fermentation technology. This poses an economic risk since it prevents the rapid growth of the fermentation industry, compelling the need to acquire native, suitable yeast strains from regional substrates to produce sustainable ethanol. Hence, the objective of this study is to identify native yeast strains from Marcha and evaluate their capacity for ethanol production.

## 2. Materials and Methods

### 2.1. Collection of Marcha Samples

The materials used in this study were collected from 10 different geographical regions in Nepal (Table S1 and Figure S1). Subsequently, these samples were transported to the laboratory and kept at a temperature of 5°C for further investigation.

### 2.2. Isolation of Yeasts

Samples of Marcha were crushed using a sterile mortar and pestle. A Marcha sample weighing 1 g was homogenized in 9 mL of 0.85% sterile physiological saline, and serial dilutions were carried out up to a dilution factor of 10^7^. Two decimal dilutions (10^6^ and 10^7^) were then grown onto yeast extract peptone (YEP) agar medium supplemented with ampicillin (100 ng *μ*L^−1^) at 28°C–30°C for 48–72 h, following the method described by Jeyaram et al. [13]. Based on the colony morphology, the representative colonies were selected and further subcultured on new YEP media plates. On YEP agar, colonial appearance, including colony size, shape, surface appearance, and pigment production, was observed. The yeast isolates that successfully passed the screening process were then stored in YEP agar slants at 5°C for further use.

### 2.3. Characterization and Identification of Isolated Yeasts

We have followed various researchers' procedures for the identification of isolated yeasts. The parameters for identification specifically include colonial and morphological characteristics [14], spore formation [15], fermentation of various sugars [16, 17], formation of the pellicle [18], flocculation ability [19], hydrogen sulfide production [20], 1% acetic acid tolerance [21], acid production [16], and amylase [22]. To differentiate between wine yeast and wild yeast, a sandwich spread culturing technique was employed [23]. A volume of 0.1 mL of the yeast isolate was spread on the solidified lower layer medium. Subsequently, the upper layer of the medium was poured on top, and the plates were then incubated at 28°C–30°C for 48–72 h. During the incubation period, the plates were observed for the presence of red-colored colonies, which helped in distinguishing between the two types of yeast.

### 2.4. Assessment of the Yeasts for Attributes Essential for Ethanol Production Screening

#### 2.4.1. Stress Exclusion Tests

The isolates were initially cultured on YPG medium at 37°C for 48 h. Those showing growth were further cultured on YPG supplemented with ethanol (80 mL/L) and incubated at 28°C–30°C for 48 h. The resulting growing isolates were then transferred to YP supplemented with glucose (200 g/L) and incubated under the same conditions [24]. Following this incubation, the colonies were cultured on YP supplemented with sucrose (200 g/L) and ethanol (80 ml/L) and incubated at the same conditions.

#### 2.4.2. Stress Tolerance Tests

The isolates were subjected to various stress tests to evaluate their ability to withstand various conditions. A small amount of the inoculum was added to 10 mL of yeast malt extract peptone dextrose (YMPD) broth containing different stress-inducing factors. These factors included varying percentages of ethanol (10%, 15%, and 20% *v*/*v*), dextrose (15% and 38% *w*/*v*), temperature (30°C, 37°C, and 45°C), pH (2.8, 3.0, and 3.2), and sulfur dioxide (100, 250, and 500 mg/L), following the methodology by Benitez, Del Castillo, and Aguilera [25]. The growth of yeast cells was monitored by comparing them to the yeast inoculated in YMPD broth without any stress parameters, which served as the control tube.

#### 2.4.3. Ethanol Production

To evaluate the yeast's alcohol-producing capacity, 1 mL of supernatant obtained from the centrifuged broth of 24-h-grown yeast isolates was combined with 200 *μ*L of potassium chromate reagent, following the method outlined by Bhatia and Paliwal [26]. The mixture, along with standard ethanol, was then subjected to boiling for 10 min. As a result of boiling, the color of the solution changed from orange to green. Subsequently, the optical density of the solution was measured at 600 nm to determine the yeast's alcohol production capability. The results are presented as the average of three consecutive experiments' outputs.

#### 2.4.4. Statistical Analysis

A chi-square test was conducted to examine the relationship between yeast groups and five different parameters (ethanol, glucose, temperature, pH, and SO_2_). The chi-square test for independence was selected due to its appropriateness for categorical data. The observed frequencies of the grouping of yeast and different parameters were compared to the expected frequencies under the null hypothesis of independence. All statistical analyses were performed using Microsoft Excel with a significance level set at 0.05.

## 3. Results

### 3.1. Characterization and Identification of Yeast From Marcha Sample

A total of 43 yeast strains were obtained from 27 samples, which showed two distinct colony characteristics. One group formed spherical, grey, and shiny colonies, while the other group formed white, smooth, and flat colonies (Table 1). Additionally, the vegetative cells were observed as spherical, oval, or elongated with single or multiple buds (Figure S2). Furthermore, 55.8% of yeast isolates can ferment all six sugars except lactose, while 30.% isolates ferment glucose, maltose, and galactose, and the remaining 14% isolates ferment glucose and galactose only (Table S2). On this sugar fermentation pattern differences, the isolates could be grouped as Groups A, B, and C. Similarly, distinct characteristic features were observed among these categorized groups. Group C showed flocculation and acetic acid tolerance abilities, whereas Group B demonstrated amylase production. Group A shared the pellicle production and hydrogen sulfide tolerance characteristics with Group C. Based on colony colors and wild and wine yeast differentiation media, both white and red colonies were observed in Group A isolates only, whereas Group B showed red colonies and Group C showed white colonies.

### 3.2. Assessment of Yeast Isolates for Attributes Essential in Industrial Fermentation of Ethanol

**Figure 1:**
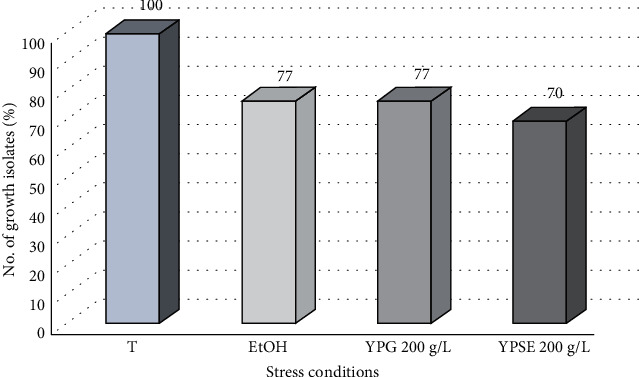
Yeast isolates from stress exclusion tests. T, temperature stress test; EtOH, ethanol stress test; YPG 200 g/L, osmotic stress test with glucose; YPSE 200 g/L, osmotic stress with sucrose and alcohol.

The yeast isolates underwent stress exclusion testing to identify potent strains for further characterization (Table S3). All 43 isolates (100%) showed growth at 37°C. When cultured on YP medium supplemented with 8% ethanol, 33 isolates (76.74%) exhibited growth. Among the ethanol-resistant isolates (*n* = 33), all of them also demonstrated resistance to osmotic stress with 20% glucose in the YP medium. However, three strains were found to be nonresistant when cultured on a YP medium supplemented with 20% sucrose and 8% alcohol. Consequently, out of 43 isolates, only 30 were able to grow under all tested conditions (Figure 1).

**Figure 2:**
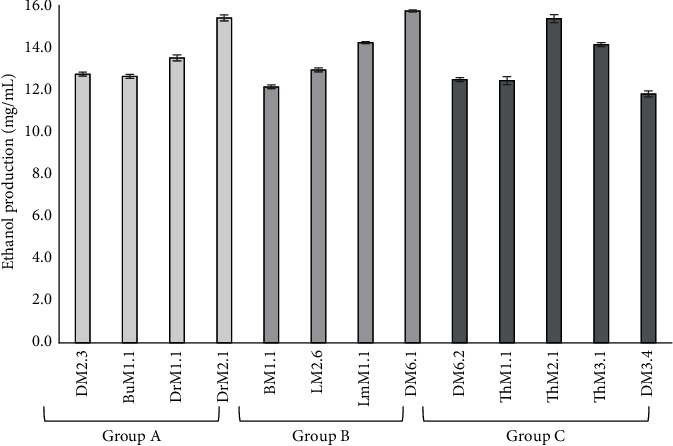
Ethanol production of potent ethanol tolerance yeast isolates. Error bars represent standard deviations from the mean of three biological samples.

Further stress tolerance tests were conducted on the isolates that had passed the stress exclusion tests. There was variation in their tolerance levels to different glucose concentrations. Almost all Group A isolates showed an intensive growth pattern in all glucose-incorporated media (24%, 30%, and 326%), whereas Groups B and C showed intensive growth up to 30% glucose-added media (Table 2). Similarly, some of the Group A isolates showed intensive growth at high temperatures (45°C), while others and Group B and C isolates retarded at this high temperature. Furthermore, all groups of isolates showed an intensive growth pattern at lower margins of ethanol- (10%) and pH- (3.8) added media. The effect of sulfur dioxide was found to be ineffective up to the concentration of 500 mg/L for most isolates of Groups A and C, whereas positive effects on Group B isolates increased with increasing concentration. Based on their ethanol tolerance growth, four isolates each of Groups A and B and five isolates of Group C were selected for ethanol production. All the yeast isolated within each group produced ethanol in the range of 12–16 mg/mL (Figure 2). The different test parameters were found to be independent for identified yeast isolates, as the calculated chi-square test is lower than the critical value (Table 3).

## 4. Discussion

The study's findings reveal that indigenous yeasts with favorable fermentation characteristics, capable of improving ethanol yield and reducing production costs, were isolated from Marcha. The identification process primarily relied on colonial and morphological characteristics, as well as their ability to assimilate specific carbon compounds. Yeasts, being microorganisms that derive ATP energy from the breakdown of organic compounds, display metabolic diversity, leading to variations in their fermentative patterns [27]. *Saccharomyces cerevisiae* has been identified as a yeast species capable of fermenting sucrose, maltose, fructose, glucose, galactose, and raffinose [19]. According to Stewart [28], both *Saccharomyces* and *Endomycopsis* strains possess the necessary mechanisms, such as energy-dependent maltose permease and maltase enzymes, to ferment maltose (a disaccharide) into two glucose units. Additionally, *Endomycopsis* strains are highly amylolytic, effectively hydrolyzing starch into sugars [29]. Consequently, the identified yeast isolates are likely to belong to the genera *Saccharomyces*, *Endomycopsis*, and *Zygosaccharomyces*. The yeast isolated from Marcha exhibited a diverse microbial population, with a significant presence of amylolytic starters based on their morphological and physiological characteristics. Similar yeast genera were also found in other Asian amylolytic starters [1, 10, 30]. Strains of *Saccharomyces* were previously identified in ragi and banh men [1, 31].

Regarding the jaanr fermentation, it is commonly believed that two types of yeast were pivotal: amylolytic yeasts from Marcha, particularly *Endomycopsis*, which degrade starch into glucose, and alcohol-producing yeasts that rapidly utilize the glucose to produce ethanol [3, 32]. The majority of Marcha and loog-pang (Thailand rice cake starter) samples contained *Endomycopsis*, which exhibited strong amylolytic activity. Although the distribution of yeast species varied among different Marcha samples, all tested samples contained both starch degraders and ethanol producers, supporting the previously proposed role of yeasts in jaanr fermentation. Among the ethanol producers, *Saccharomyces* and *Endomycopsis* strains showed the highest ethanol productivity, comparable to wine yeast. *Saccharomyces* strains were dominant in specific jaanr samples (kodo ko jaanr and makai ko jaanr; unpublished data), suggesting that closely related species within the *Saccharomyces* genus play a central role in alcohol fermentation.

Evaluating how yeast strains respond to various stress conditions provides valuable insights into their adaptability and performance as impaired yeast during fermentation. Impaired yeast refers to strains that do not thrive optimally under fermentation conditions and face continuous exposure to different stresses, notably osmotic and ethanol stress [33, 34]. Previous research suggests that many *Saccharomyces* strains isolated from traditional fermentation processes have developed physiological adaptations to cope with extreme conditions [35]. In this study, the 43 isolated yeast strains underwent stress testing to identify those best suited for industrial applications. It was observed that all strains resistant to ethanol stress also displayed resistance to osmotic stress induced by glucose. This resistance likely correlates with specific gene expressions and could pose challenges during the fermentation process [36]. Additionally, approximately 70% of the isolates (*n* = 30) exhibited resilience to osmotic stress caused by sucrose. This resistance is a critical indicator of high invertase activity, a crucial trait for wine production [37].

The yeast strain demonstrated desirable traits, including its ability to flocculate, withstand osmotic and thermal stresses, and efficiently ferment higher concentrations of sugar, leading to significant ethanol production. These favorable characteristics make the strain a promising candidate for industrial ethanol production [38]. The flocculation ability refers to the yeast cells' capability to stick together, facilitating easy separation from the fermentation broth. This feature has economic implications as it reduces the energy expenditure involved in biomass centrifugation during yeast production [39]. The high thermotolerance observed in certain yeast strains can be attributed to the expression of heat shock proteins (Hsps), which act as molecular chaperones. These proteins play an essential role in protein synthesis, folding, trafficking, maturation, and degradation. They are swiftly induced in response to elevated temperatures, offering protection and aiding in the survival of cells [40–42]. Regarding ethanol tolerance, the variations among the yeast strains align with the findings reported by Gibson et al. [43]. The study highlights that stress induced by increasing ethanol concentrations during fermentation can result in toxic levels of ethanol accumulation, leading to reduced ethanol production and potentially stalled fermentation. These findings emphasize the importance of identifying yeast strains with strong ethanol tolerance for efficient ethanol production. Moreover, according to Benitez, Del Castillo, and Aguilera [25], wine yeasts exhibit robust growth at 10% ethanol concentration. During yeast cell proliferation, they create an acidic extracellular environment due to plasma membrane H^+^-ATPase activity and the release of acidic metabolites. The protein kinase C (PKC) pathway plays a vital role in maintaining cell shape and integrity in *Saccharomyces cerevisiae*, specifically conferring tolerance to low pH levels. Essential proteins within this pathway, like BcK1 and Slt2 from the mitogen-activated protein kinase cascade, ensure cell survival under acidic conditions. Consequently, most yeast isolates have shown growth under acidic conditions and hydrogen sulfide production abilities, which provides valuable insights into their ability to cope with potentially toxic levels of SO_2_ in the environment. This knowledge is crucial as it determines the yeast's survival capabilities and its capacity to outcompete other microorganisms for essential nutrients [44]. However, it is essential to note that high levels of hydrogen sulfide production by yeasts are undesirable in wine production due to the unwanted flavors and tastes they impart, potentially compromising the wine's overall quality [45].

The identification of yeast species traditionally relies on evaluating 70–90 tests based on morphology, biochemical characteristics, and sexual reproduction [46]. While this process is well established, it is not entirely definitive. Due to the high mutation rates in wild yeasts, molecular techniques for characterizing and analyzing polymorphisms are being developed [47]. Therefore, future research is aimed at integrating both traditional and molecular methods to efficiently identify yeasts with more robust characteristics.

## 5. Conclusion

In the present study, there was the involvement of different yeasts in Marcha. In morphological characterization, all the identified yeasts showed different colony and cell characteristics regarding color (white or grey), shape (circular or irregular), and elevation (flat or raised) on the YMA plates.

In physiological characterization, the identified yeasts were able to grow at the optimal growth temperature, pH, and ethanol concentration ranging from 30°C to 37°C, 3 to 3.8, and 10%–13%. In biochemical characterization, all identified isolates of yeast were densely grown and fermented in monosaccharides rather than disaccharides and polysaccharides sugars. Some of the yeast isolates were flocs, amylase, and hydrogen sulfide producers, which are the potential attributes for ethanol fermentation. Finally, three dominant groups of yeasts could be identified from Marcha samples from 10 different geographic belts.

## Figures and Tables

**Table 1:** Morphological characteristics of the isolates.

**S. no.**	**Macroscopic characteristics**		**Microscopic characteristics**		**No. of isolates**
	**Color and shape**	**Surface appearances**	**Cell shape**	**Arrangement**	
1	White and spherical	Smooth and flat	Spherical or elongated	Single or multipolar budding	19
2	Creamy and spherical	Smooth, shiny, flat, or raised	Spherical, elongated, or oval	Single or multipolar budding	24

**Table 2:** Attributes of the yeast strains isolated from Marcha for ethanol production.

**Strain**	**Isolate**	**Ethanol (%, ** **v**/**v)**			**Glucose (%, ** **w**/**v)**			**Temp. (°C)**			**pH**			**SO **_**2**_ ** (mg/L)**		
		**10**	**13**	**15**	**24**	**30**	**36**	**30**	**37**	**45**	**2.8**	**3**	**3.8**	**100**	**250**	**500**
Group A	BM1.2	++	−	−	+++	+++	+++	+++	+++	+++	−	−	+	++	++	−
	LM2.5	++	−	−	+++	+++	+++	+++	+++	−	−	++	++	++	+	+
	LM3.2	++	−	−	+++	+++	+++	+++	+++	+	+	+	+	+++	++	++
	DhM1.5	+++	−	−	+++	+++	+++	+++	+++	++	++	++	+++	+++	++	++
	DhM1.6	−	−	−	+++	+++	++	+++	+++	−	−	−	+	++	+	−
	DhM2.6	++	+	−	+++	+++	++	+++	+++	++	+	++	+++	+++	+++	++
	DM1.6	+	−	−	++	+++	++	+++	+++	−	−	−	++	+++	+++	++
	DM2.3	+++	+	−	++	+++	++	++	++	−	+	+	++	+++	+++	++
	BuM1.1	++	+	−	+++	+	+	+++	+++	−	−	−	−	+++	+++	++
	BuM2.1	++	−	−	+++	+++	++	+++	+++	−	+	++	+++	+++	++	++
	DrM1.1	+++	++	−	+++	+++	++	+++	+++	−	−	−	−	+++	++++	+++
	DrM2.1	+++	+++	++	+++	+++	+++	+++	+++	+++	++	++	+++	+++	+++	+++
	ThM2.2	+	−	−	++	+++	++	++	++	−	−	−	++	+++	++	++

Group B	BM1.1	++	+	−	++	+++	++	++	++	−	+	+	+++	+++	++	+
	LM1.1	++	−	−	+++	+++	++	+++	+++	−	−	−	++	+++	++	+
	LM2.6	+++	++	−	+++	+++	+++	+++	+++	−	−	++	++	+++	++	++
	DhM2.5	++	−	−	+++	+++	++	+++	++	−	−	−	++	+++	+++	++
	LmM1.1	+++	++	−	+++	+++	+++	+++	+++	−	+	+	++	+	−	−
	LmM1.5	−	−	−	++	++	+	+++	++	−	−	−	++	+	−	−
	LmM1.6	−	−	−	++	+	−	++	++	−	−	−	++	+	−	−
	LmM2.2	−	−	−	++	+	−	++	++	−	−	+	++	++	+	+
	DM6.1	+++	+++	++	+++	+++	++	+++	+++	−	−	−	++	+++	+++	

Group C	DM6.2	+++	+	−	++	++	+	+++	+++	−	−	−	++	+++	+++	++
	ThM1.1	+++	++	−	+++	+++	++	+++	+++	−	+	+	++	+++	++	++
	ThM2.1	+++	+++	++	+++	+++	+++	+++	+++	−	+	++	+++	+++	++	++
	ThM3.1	+++	++	−	+++	+++	++	+++	+++	−	+	+	++	+++	++	+
	BM2.5	++	−	−	++	++	+	+++	+++	−	−	−	+	+++	++	+
	DM3.4	++	+	−	++	++	+	+++	+++	−	+	++	+++	+++	++	++
	BhM2.2	−	−	−	++	+	−	++	++	−	−	−	++	+++	++	++
	SM2	−	−	−	+	+	−	++	++	−	+	+	++	+++	+++	++

Note: −: no growth; +: low growth; ++: moderate growth; +++: intensive growth.

Abbreviations: B, Banepa; Bh, Bhaktapur; Bu, Butwal; D, Dang; Dh, Dhading; Dr, Dharan; L, Lubu; Lm, Lamjung; M, Marcha; S, Syanja; Temp., temperature; Th, Thimi.

**Table 3:** Chi-square test for the determination of correlation between different parameters and yeast isolation.

	**Observed (expected value/critical value)**			
	**Group A**	**Group B**	**Group C**	**Chi-square**
Ethanol	13.79 (18.04)	10.34 (9.25)	13.79 (10.64)	9.53 (15.507^a^)
Glucose	34.48 (29.52)	17.24 (15.14)	10.34 (17.41)	
Temperature	37.93 (37.72)	20.68 (19.34)	20.68 (22.24)	
pH	13.79 (11.48)	3.44 (5.88)	6.89 (6.77)	
SO_2_	34.48 (37.72)	17.24 (19.34)	27.31 (22.24)	

*Note:* Observed data: the data were retrieved from Table 2 taken of those isolates that show intensive growth at lower margins for different stress parameters.

^a^Critical value at *p* > 0.05.

## Data Availability

Data will be made available on request.

## Supporting Information

Supporting Information Additional supporting information can be found online in the Supporting Information section. Table S1: list of the Marcha samples from different parts of Nepal. Table S2: characterization and identification of yeast isolates. Table S3: stress exclusion test of isolated yeasts. Figure S1: geographical distribution of Marcha of Nepal. Figure S2: microscopic observation of yeast isolates under 40x magnification.

## References

[1] Hesseltine C. W., Rogers R., Winarno F. G. (1988). Microbiological studies on amylolytic oriental fermentation starters. *Mycopathologia*.

[2] Park K. I., Mheen T. I., Lee K. H., Chang C. H., Lee S. R., Kwon T. W. (1977). Korean yakju and takju. *The Proceeding and Symposium on Indigenous Fermented Foods*.

[3] Tamang J. P., Sarkar P. K. (1995). Microflora of murcha: an amylolytic fermentation starter. *Microbios*.

[4] Heard G. M., Fleet G. H. (1988). The effects of temperature and pH on the growth of yeast species during the fermentation of grape juice. *Journal of Applied Bacteriology*.

[5] Fleet G. H. (2003). Yeast interactions and wine flavor. *International Journal of Food Microbiology*.

[6] Lambrechts M. G., Pretorius I. S. (2000). Yeast and its importance to wine aroma: a review. *South African Journal of Enology and Viticulture*.

[7] Romano P., Fiore C., Paraggio M., Caruso M., Capece A. (2003). Function of yeast species and strains in wine flavour. *International Journal of Food Microbiology*.

[8] Longo E., Cansado J., Agrelo D., Villa T. G. (1991). Effect of climatic conditions on yeast diversity in grape musts from Northwest Spain. *American Journal of Enology and Viticulture*.

[9] Pretorius I. S., van der Westhuizen T. J., Augustyn O. P. H. (1999). Yeast biodiversity in vineyards and wineries and its importance to the South African wine industry: a review. *South African Journal of Enology & Viticulture*.

[10] Hesseltine P., Kurtzman W. (1990). *Yeasts in Amylolytic Food Starters*.

[11] Shah S. P., Jani K., Sharma A., Pradhan P., Shouche Y., Tamag J. P. (2017). Analysis of bacterial and fungal communities in *marcha* and *thiat*, traditionally prepared amylolytic starters of India. *Scientific Reports*.

[12] Stewart G. G., Panchal C. J., Russell I., Sills A. M. (1983). Biology of ethanol-producing microorganisms. *Critical Reviews in Biotechnology*.

[13] Jeyaram K., Singh W. M., Capece A., Romano P. (2008). Molecular identification of yeast species associated with “Hamei”–a traditional starter used for rice wine production in Manipur, India. *International Journal of Food Microbiology*.

[14] Noroul Asyikeen Z., Ma’aruf A. G., Sahilah A. M., Mohd Khan A., Wan Aida W. M. (2013). A new source of Saccharomyces cerevisiae as a leavening agent in bread making. *International Food Research Journal*.

[15] Yarrow D. (1998). Chapter 11–methods for the isolation, maintenance and identification of yeasts. *The Yeasts (Fourth Edition)*.

[16] Ameh J. B., Okagbue R. N., Ahmad A. (1990). Isolation and characterisation of local yeast strains for ethanol production. *Nigeria Journal of Technical Research*.

[17] Oke T. A., Ijebor J. A. (1997). Compartive studies of yeast isolates of “burukutu” and palmwine in table wine production using orange. *Nigerian Journal of Biotechnology*.

[18] Jimoh S. O. (2012). *Comparative Molecular Analysis of Ethanol-Producing Saccharomyces Cerevisiae Strains Isolated from Fermented Beverages*.

[19] Guimarães T. M., Moriel D. G., Machado I. P., Fadel Picheth C. M. T., Bonfim T. M. B. (2006). Isolation and characterization of Saccharomyces cerevisiae strains of winery interest. *Revista Brasileira de Ciências Farmacêuticas*.

[20] Ono B. I., Ishii N., Fujino S., Aoyama I. (1991). Role of hydrosulfide ions (HS-) in methylmercury resistance in Saccharomyces cerevisiae. *Applied and Environmental Microbiology*.

[21] Middelhoven W. (2002). *Hansenula Polymorphabiology and Applications*.

[22] Fossi B. T., Tavea F., Ndjouenkeu R. (2005). Production and partial characterization of a thermostable amylase from ascomycetes yeast strain isolated from starchy soils. *African Journal of Biotechnology*.

[23] Association of Official Analytical Chemists (AOAC) (1990). *Official Methods of Analysis of the AOAC, 15th ed., Methods 932.06, 925.09, 985.29, 923.03*.

[24] Umeh S. O., Agwuna L. C., Okafor U. C. (2017). Yeasts from local sources: an alternative to the conventional brewer’s yeast. *International Journal of Biotechnology and Food Science*.

[25] Benitez T., Del Castillo L., Aguilera A. (1983). Selection of wine yeasts for growth and fermentation in the presence of ethanol and sucrose. *Applied and Environmental Microbiology*.

[26] Bhatia L., Paliwal S. (2010). Banana peel waste as substrate for ethanol production. *International Journal of Biotechnology and Bioengineering Research*.

[27] Creveny K. L., McCaffery J. M., Jensen R. E. (2001). Division of mitochondria requires a NovelDNM1-interacting protein, Net2p. *Molecular Biology of the Cell*.

[28] Stewart J. D. (2006). Genomes as resources for biocatalysis. *Advances in Applied Microbiology*.

[29] Chaowsungket M. (1978). *Selection of Yeast and Mould Strains for Rice Wine Production, [M.S. thesis]*.

[30] Deák T. (1991). Foodborne yeasts. *Advances in Applied Microbiology*.

[31] Lee A. C., Fujio Y. (1999). Microflora of banh men, a fermentation starter from Vietnam. *World Journal of Microbiology and Biotechnology*.

[32] Thapa S. (2001). *Microbiological and Biochemical Studies of Indigenous Fermented Cereal-Based Beverages of the Sikkim Himalayas, [Ph.D. thesis],*.

[33] Ivorra C., Pérez‐Ortín J. E., del Olmo M. (1999). An inverse correlation between stress resistance and stuck fermentations in wine yeasts. A molecular study. *Biotechnology and Bioengineering*.

[34] Querol A., Fernández-Espinar M. T., Del Olmo M., Barrio E. (2003). Adaptive evolution of wine yeast. *International Journal of Food Microbiology*.

[35] Pataro C., Guerra J. B., Petrillo-Peixoto M. L., Mendonça-Hagler L. C., Linardi V. R., Rosa C. A. (2000). Yeast communities and genetic polymorphism of Saccharomyces cerevisiae strains associated with artisanal fermentation in Brazil. *Journal of Applied Microbiology*.

[36] Zuzuarregui A., del Olmo M. (2004). Analyses of stress resistance under laboratory conditions constitute a suitable criterion for wine yeast selection. *Antonie van Leeuwenhoek*.

[37] Pataro C., Santos A., Correa S. R., Morais P. B., Linardi V. R., Rosa C. A. (1998). Physiological characterization of yeasts isolated from artisanal fermentation in an *aguardente* distillery. *Revista de Microbiologia*.

[38] Brooks A. A. (2008). Ethanol production potential of local yeast strains isolated from ripe banana peels. *African Journal of Biotechnology*.

[39] Nahvi I., Emtiazi G., Alkabi L. (2002). Isolation of a flocculating Saccharomyces cerevisiae and investigation of its performance in the fermentation of beet molasses to ethanol. *Biomass and Bioenergy*.

[40] Young J. C., Agashe V. R., Siegers K., Hartl F. U. (2004). Pathways of chaperone-mediated protein folding in the cytosol. *Nature reviews Molecular Cell Biology*.

[41] Burnie J. P., Carter T. L., Hodgetts S. J., Matthews R. C. (2006). Fungal heat-shock proteins in human disease. *FEMS Microbiology Reviews*.

[42] Westerheide S. D., Morimoto R. I. (2005). Heat shock response modulators as therapeutic tools for diseases of protein conformation. *Journal of Biological Chemistry*.

[43] Gibson B. R., Lawrence S. J., Leclaire J. P. R., Powell C. D., Smart K. A. (2007). Yeast responses to stresses associated with industrial brewery handling. *FEMS Microbiology Reviews*.

[44] Park H., Bakalinsky A. T. (2000). *SSU1* mediates sulphite efflux in Saccharomyces cerevisiae. *Yeast*.

[45] Ribeiro C. A. F., Horii J. (1999). Potencialidades de linhagens de levedura Saccharomyces cerevisiae para a fermentação do caldo de cana. *Scientia Agrícola*.

[46] Deak T. (1995). Methods for the rapid detection and identification of yeasts in foods. *Trends in Food Science & Technology*.

[47] Song H. T., Liu S. H., Gao Y. (2016). Simultaneous saccharification and fermentation of corncobs with genetically modified Saccharomyces cerevisiae and characterization of their microstructure during hydrolysis. *Bioengineered*.

