# Supplementary material for: Isolation and Identification of Yeasts in Marcha, a Rice Wine Starter Culture From Nepal
Source: Int J Food Sci. 2024 Sep 16;2024:4188578. doi: 10.1155/2024/4188578 (PMC11419841; doi:10.1155/2024/4188578)
Supplement: Supporting Information — Additional supporting information can be found online in the Supporting Information section. Table S1: list of the Marcha samples from different parts of Nepal. Table S2: characterization and identification of yeast isolates. Table S3: stress exclusion test of isolated yeasts. Figure S1: geographical distribution of Marcha of Nepal. Figure S2: microscopic observation of yeast isolates under 40x magnification. [file 4188578.f1.docx]

**Isolation and identification of yeasts in marcha, a rice starter culture from Nepal**

Jayram Karmacharya^1*^, Prasansah Shrestha^1^, Tika Bahadur Karki^1^_,_ Om Prakash Pant^1,2^

^1^Department of Microbiology, National College (NIST), Tribhuvan University, P.O. Box: 8659, Naya Bazar, Nepal

^2^Central Campus of Technology, Tribhuvan University, Dharan, Nepal

*Correspondence: [jayram.karmacharya@gmail.com](mailto:jayram.karmacharya@gmail.com) (J.K)

**Table S1.** List of the Marcha samples from different parts of Nepal

| **S. No.** | **Sampling area** | **No. of samples** | **Sample code** |
| --- | --- | --- | --- |
| 1 | Banepa | 2 | BM1, BM2 |
| 2 | Lamjung | 2 | LmM1, LmM2 |
| 3 | Lubu | 4 | LM1, LM2, LM3, LM4 |
| 4 | Dhading | 2 | DhM1, DhM2 |
| 5 | Daang | 6 | DM1, DM2, DM3, DM4, DM5, DM6 |
| 6 | Dharan | 2 | DrM1, DrM2 |
| 7 | Thimi | 3 | ThM1, ThM2, ThM3 |
| 8 | Bhaktapur | 2 | BhM1, BhM2 |
| 9 | Syanja | 2 | SM1, SM2 |
| 10 | Butwal | 2 | BuM1, BuM2 |

**B**: Banepa, **Lm**: Lamjung, **L**:Lubu, **Dh**: Dhading, **D**: Dang, **Dr**: Dharan, **Th**: Thimi, **Bh**: Bhaktapur, **S**: Syanja, **Bu**: Butwal, **M**: Marcha

**Table S2.** Characterization and identification of yeast isolates

| **Isolate** | **Fermentation** | | | | | | **Pel.** | **Flocc.** | **Ascop.** | **Acid** | **WYDM**  **growth** | **Amylase** | **H_2_S** | **Acetic acid** | **Identified** |
| --- | --- | --- | --- | --- | --- | --- | --- | --- | --- | --- | --- | --- | --- | --- | --- |
|  | **Lac** | **Gal** | **Raf** | **Mal** | **Suc** | **Glu** |  |  |  |  |  |  |  |  |  |
| BM1.1 |  |  |  |  |  |  |  |  |  |  |  |  |  |  |  |
| LM1.1 |  |  |  |  |  |  |  |  |  |  |  |  |  |  |  |
| LM2.6 |  |  |  |  |  |  |  |  |  |  |  |  |  |  |  |
| DhM2.5 |  |  |  |  |  |  |  |  |  |  |  |  |  |  |  |
| LmM1.1 |  |  |  |  |  |  |  |  |  |  |  |  |  |  |  |
| LmM1.5 |  |  |  |  |  |  |  |  |  |  |  |  |  |  |  |
| LmM1.6 |  |  |  |  |  |  |  |  |  |  |  |  |  |  |  |
| LmM2.2 | **-** | **+** | **-** | **+** | **-** | **+** | - | - | - | + | Red | + | + | - | Group B |
| DM6.1 |  |  |  |  |  |  |  |  |  |  |  |  |  |  |  |
| DM6.2 |  |  |  |  |  |  |  |  |  |  |  |  |  |  |  |
| ThM1.1 |  |  |  |  |  |  |  |  |  |  |  |  |  |  |  |
| ThM2.1 |  |  |  |  |  |  |  |  |  |  |  |  |  |  |  |
| ThM3.1 |  |  |  |  |  |  |  |  |  |  |  |  |  |  |  |
| BM2.5 |  |  |  |  |  |  |  |  |  |  |  |  |  |  |  |
| BM2.6 |  |  |  |  |  |  |  |  |  |  |  |  |  |  |  |
| LM3.3 | **-** | **+** | **-** | **-** | **-** | **+** | + | + | - | + | White |  | +++ | + | Group C |
| DM3.4 |  |  |  |  |  |  |  |  |  |  |  |  |  |  |  |
| BhM2.2 |  |  |  |  |  |  |  |  |  |  |  |  |  |  |  |
| SM2 |  |  |  |  |  |  |  |  |  |  |  |  |  |  |  |

**Lc**: lactose, **Gal**: galactose, **Raf**: raffinose, M**al**: maltose, **Suc**: sucrose, **Glu**: glucose; **Pel**.: pellicle formation, **Flocc**.: floccule formation, **Ascop**.: ascospore formation, **WYDM:** Wine yeast differentiating media, **Red**: red-colored colonies, **White**: white-coloured colonies, **Amylase**: amylase production, **H_2_S**: H_2_S production, **Acetic acid**: 1% acetic acid tolerance. **Note**: (+): low growth, (++): moderate growth, (+++): intensive growth, (-): no growth.

**Table S2.** Characterization and identification of yeast isolates (continued)

| **Isolate** | **Fermentation** | | | | | | **Pel.** | **Flocc.** | **Ascop.** | **Acid** | **WYDM**  **growth** | **Amylase** | **H_2_S** | **Acetic acid** | **Identified** |
| --- | --- | --- | --- | --- | --- | --- | --- | --- | --- | --- | --- | --- | --- | --- | --- |
|  | **Lac** | **Gal** | **Raf** | **Mal** | **Suc** | **Glu** |  |  |  |  |  |  |  |  |  |
| BM1.2 |  |  |  |  |  |  |  |  |  |  |  |  |  |  |  |
| LM2.5 |  |  |  |  |  |  |  |  |  |  |  |  |  |  |  |
| LM3.2 |  |  |  |  |  |  |  |  |  |  |  |  |  |  |  |
| LM4.1 |  |  |  |  |  |  |  |  |  |  |  |  |  |  |  |
| DhM1.5 |  |  |  |  |  |  |  |  |  |  |  |  |  |  |  |
| DhM1.6 |  |  |  |  |  |  |  |  |  |  |  |  |  |  |  |
| DhM2.6 |  |  |  |  |  |  |  |  |  |  |  |  |  |  |  |
| LmM1.2 |  |  |  |  |  |  |  |  |  |  |  |  |  |  |  |
| LmM2.3 |  |  |  |  |  |  |  |  |  |  |  |  |  |  |  |
| DM1.5 |  |  |  |  |  |  |  |  |  |  |  |  |  |  |  |
| DM1.6 |  |  |  |  |  |  |  |  |  |  |  |  |  |  |  |
| DM2.2 | **-** | **+** | **+** | **+** | **+** | **+** | + | - | - | + | Red/  White | - | +++ | - | Group A |
| DM2.3 |  |  |  |  |  |  |  |  |  |  |  |  |  |  |  |
| DM3.5 |  |  |  |  |  |  |  |  |  |  |  |  |  |  |  |
| DM4.2 |  |  |  |  |  |  |  |  |  |  |  |  |  |  |  |
| DM4.3 |  |  |  |  |  |  |  |  |  |  |  |  |  |  |  |
| DM5.1 |  |  |  |  |  |  |  |  |  |  |  |  |  |  |  |
| BhM2.3 |  |  |  |  |  |  |  |  |  |  |  |  |  |  |  |
| BuM1.1 |  |  |  |  |  |  |  |  |  |  |  |  |  |  |  |
| BuM2.1 |  |  |  |  |  |  |  |  |  |  |  |  |  |  |  |
| DrM1.1 |  |  |  |  |  |  |  |  |  |  |  |  |  |  |  |
| DrM2.1 |  |  |  |  |  |  |  |  |  |  |  |  |  |  |  |
| ThM2.2 |  |  |  |  |  |  |  |  |  |  |  |  |  |  |  |
| SM1.1 |  |  |  |  |  |  |  |  |  |  |  |  |  |  |  |

**Lc**: lactose, **Gal**: galactose, **Raf:** raffinose, **Mal**: maltose, **Suc**: sucrose, **Glu**: glucose; **Pel**.: pellicle formation, **Flocc**.: floccule formation, **Ascop**.: ascospore formation, **WYDM**: Wine yeast differentiating media, **Red**: red-colored colonies, **White**: white-coloured colonies, **Amylase**: amylase production, **H_2_S**: H_2_S production, **Acetic acid**: 1% acetic acid tolerance. **Note**: (+): low growth, (++): moderate growth, (+++): intensive growth, (-): no growth.

**Table S3.** Stress exclusion test of isolated yeasts

| **Isolate code** | **YPG at 37^0^ C** | **YPG supp alcohol (8%)** | **YPG supp glucose (20%)** | **YPG supp (sucrose & alcohol)** |
| --- | --- | --- | --- | --- |
| LmM1.1 | + | + | + | + |
| LmM1.2 | + | - | NF | NF |
| LmM1.5 | + | + | + | + |
| LmM1.6 | + | + | + | + |
| LmM2.2 | + | + | + | + |
| LmM2.3 | + | + | + | - |
| DM1.5 | + | + | + | - |
| DM1.6 | + | + | + | + |
| DM2.2 | + | - | NF | NF |
| DM2.3 | + | + | + | + |
| DM3.4 | + | + | + | + |
| DM3.5 | + | - | NF | NF |
| DM4.2 | + | - | NF | NF |
| DM4.3 | + | - | NF | NF |
| DM5.1 | + | - | NF | NF |
| DM6.1 | + | + | + | + |
| DM6.2 | + | + | + | + |
| BhM2.2 | + | + | + | + |
| BhM2.3 | + | + | + | - |
| BuM1.1 | + | + | + | + |
| BuM2.1 | + | + | + | + |
| DrM1.1 | + | + | + | + |
| DrM2.1 | + | + | + | + |
| ThM1.1 | + | + | + | + |
| ThM2.1 | + | + | + | + |
| ThM2.2 | + | + | + | + |
| ThM3.1 | + | + | + | + |
| SM1.1 | + | - | NF | NF |
| SM2 | + | + | + | + |

**Supp.**: supplement. Note : (+): growth, (-): no growth, NF: not tested further

**Table S3.** Stress exclusion test of isolated yeasts (continued)

| **Isolate code** | **YPG at 37^0^ C** | **YPG supp + alcohol (8%)** | **YPG supp + glucose (20%)** | **YPG supp + sucrose + alcohol** |
| --- | --- | --- | --- | --- |
| BM1.1 | + | + | + | + |
| BM1.2 | + | + | + | + |
| BM2.5 | + | + | + | + |
| BM2.6 | + | - | NF | NF |
| LM1.1 | + | + | + | + |
| LM2.5 | + | + | + | + |
| LM2.6 | + | + | + | + |
| LM3.2 | + | + | + | + |
| LM3.3 | + | - | NF | NF |
| LM4.1 | + | - | NF | NF |
| DhM1.5 | + | + | + | + |
| DhM1.6 | + | + | + | + |
| DhM2.5 | + | + | + | + |
| DhM2.6 | + | + | + | + |

**Supp.**: supplement. Note : (+): growth, (-): no growth, NF: not tested further


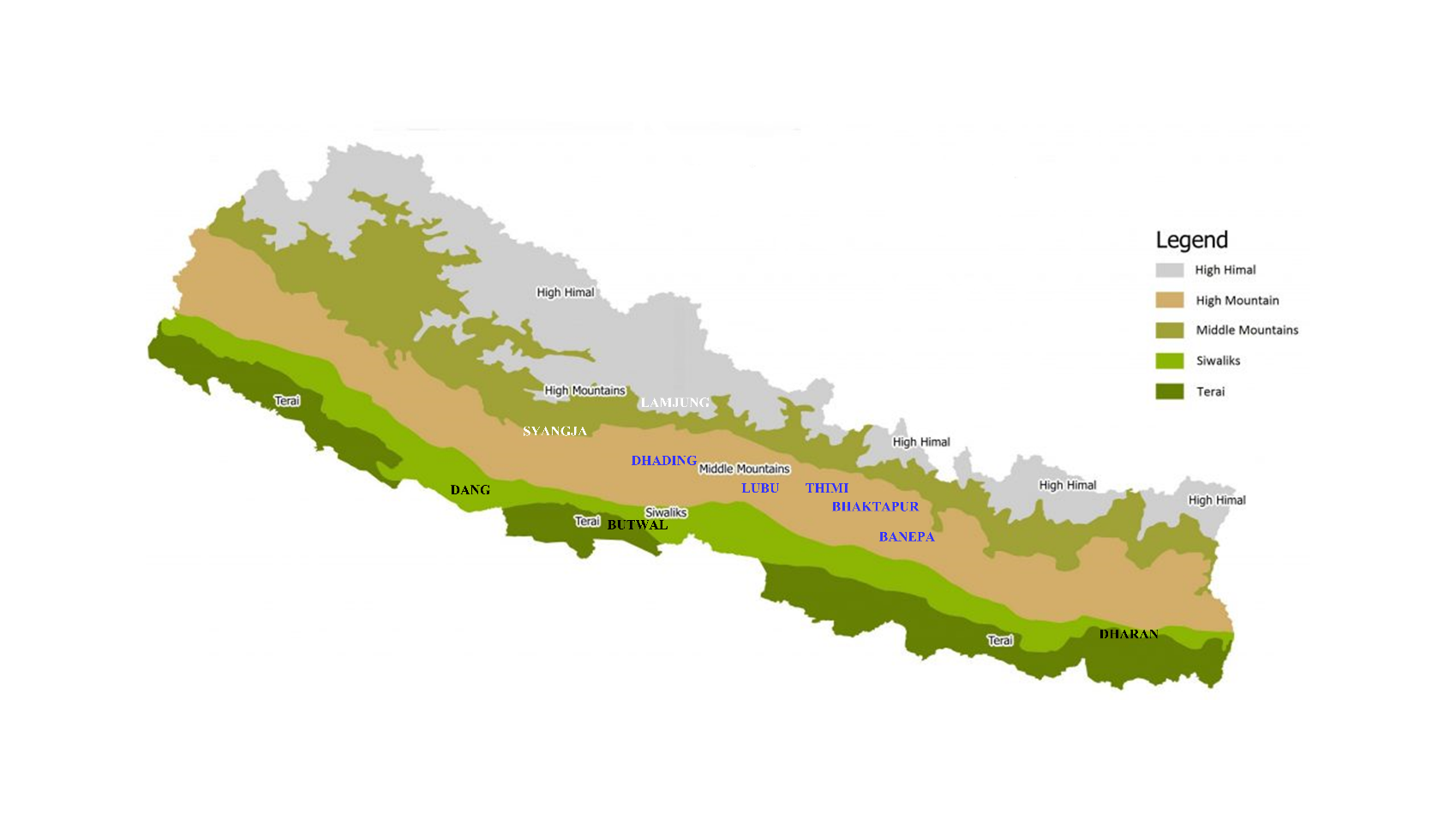


**Fig S1.** Geographical distribution of Marcha of Nepal. The Marcha samples were collected from the different belts i.e., two places in the high mountains indicated (Syangja and Lamjung; white colored), five places in the middle mountains (Dhading, Lubu, Thimi, Bhaktapur and Banepa; blue colored), two places in Siwaliks ( Dang and Dharan; black colored) and one place in Terai (Butwal; black colored).


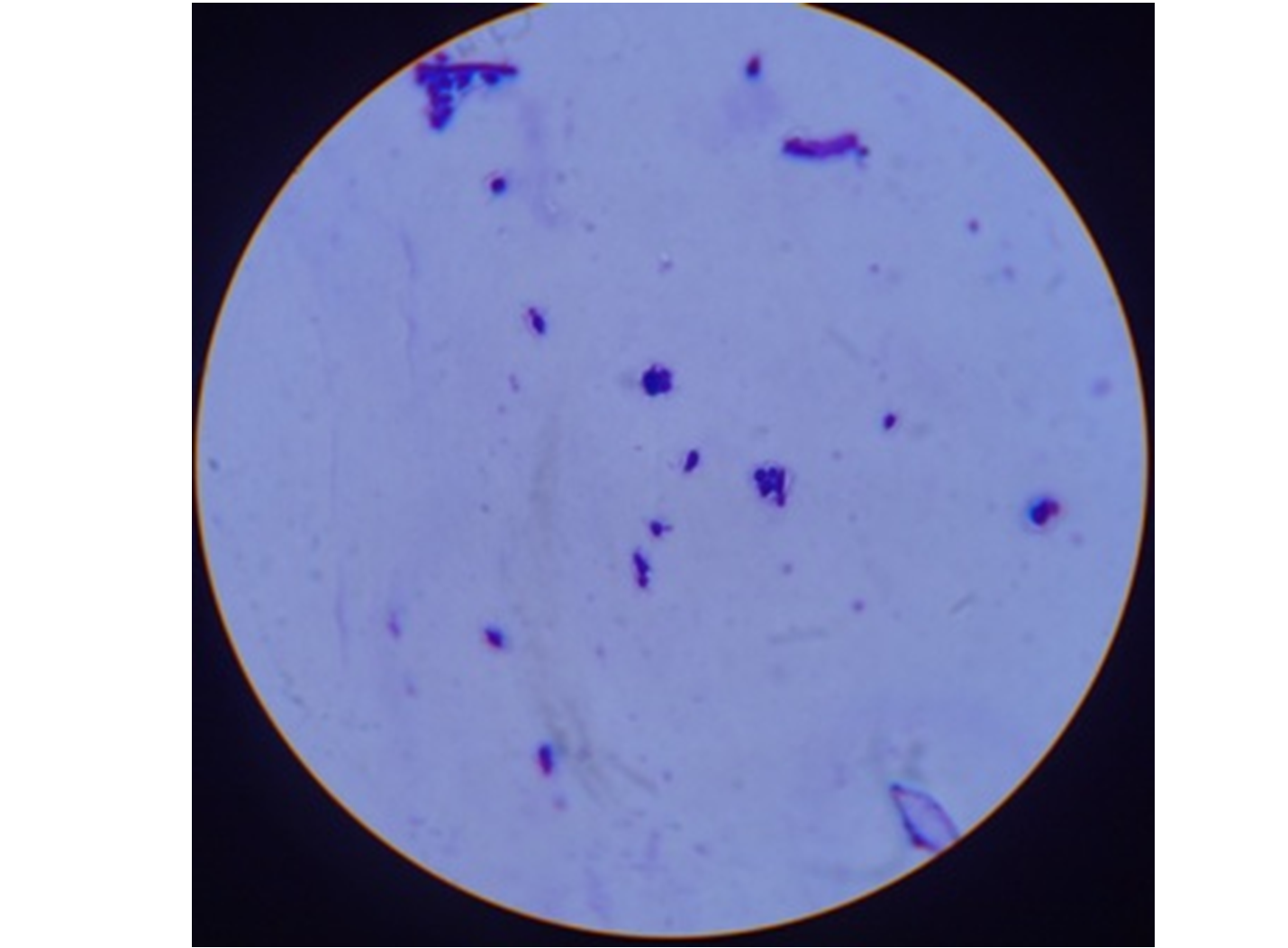


**Fig S2.** Microscopic observation of yeast isolates under 40X magnification. Yeast cells are arranged in single or multipolar budding stages.
